# Effect of Acupuncture Stimulation of Hegu (LI4) and Taichong (LR3) on the Resting-State Networks in Alzheimer's Disease: Beyond the Default Mode Network

**DOI:** 10.1155/2021/8876873

**Published:** 2021-03-08

**Authors:** Shaozhen Ji, Hao Zhang, Wen Qin, Ming Liu, Weimin Zheng, Ying Han, Haiqing Song, Kuncheng Li, Jie Lu, Zhiqun Wang

**Affiliations:** ^1^Department of Neurology, Dongfang Hospital, Beijing University of Chinese Medicine, Beijing 100078, China; ^2^Department of Radiology, Dongfang Hospital, Beijing University of Chinese Medicine, Beijing 100078, China; ^3^Department of Radiology and Tianjin Key Laboratory of Functional Imaging, Tianjin Medical University General Hospital, Tianjin 300052, China; ^4^Department of Radiology, Aerospace Center Hospital, Beijing 100049, China; ^5^Department of Neurology, Xuanwu Hospital of Capital Medical University, Beijing 100053, China; ^6^Department of Radiology, Xuanwu Hospital of Capital Medical University, Beijing 100053, China

## Abstract

It was reported that acupuncture could treat Alzheimer's disease (AD) with the potential mechanisms remaining unclear. The aim of the study is to explore the effect of the combination stimulus of Hegu (LI4) and Taichong (LR3) on the resting-state brain networks in AD, beyond the default network (DMN). Twenty-eight subjects including 14 AD patients and 14 healthy controls (HCs) matched by age, gender, and educational level were recruited in this study. After the baseline resting-state MRI scans, the manual acupuncture stimulation was performed for 3 minutes, and then, another 10 minutes of resting-state fMRI scans was acquired. In addition to the DMN, five other resting-state networks were identified by independent component analysis (ICA), including left frontal parietal network (lFPN), right frontal parietal network (rFPN), visual network (VN), sensorimotor network (SMN), and auditory network (AN). And the impaired connectivity in the lFPN, rFPN, SMN, and VN was found in AD patients compared with those in HCs. After acupuncture, significantly decreased connectivity in the right middle frontal gyrus (MFG) of rFPN (*P* = 0.007) was identified in AD patients. However, reduced connectivity in the right inferior frontal gyrus (IFG) (*P* = 0.047) and left superior frontal gyrus (SFG) (*P* = 0.041) of lFPN and some regions of the SMN (the left inferior parietal lobula (*P* = 0.004), left postcentral gyrus (PoCG) (*P* = 0.001), right PoCG (*P* = 0.032), and right MFG (*P* = 0.010)) and the right MOG of VN (*P* = 0.003) was indicated in HCs. In addition, after controlling for the effect of acupuncture on HCs, the functional connectivity of the right cerebellum crus I, left IFG, and left angular gyrus (AG) of lFPN showed to be decreased, while the left MFG of IFPN and the right lingual gyrus of VN increased in AD patients. These findings might have some reference values for the interpretation of the combination stimulus of Hegu (LI4) and Taichong (LR3) in AD patients, which could deepen our understanding of the potential mechanisms of acupuncture on AD.

## 1. Introduction

Alzheimer's disease (AD) is the most common type of dementia and a progressive neurodegenerative disease, which is characterized by two mainly pathological changes including amyloid-beta plaques and neurofibrillary tangles, finally resulting in neuronal degeneration and loss [1, 2]. The disease affected millions of elderly subjects worldwide causing remarkable costs to the society. However, there is no effective method for early diagnosis and treatment of AD in the world [3]. Acupuncture, an alternative and complementary treatment of traditional Chinese medicine, to date, has been widely used to ameliorate impairments in neuropsychiatric symptoms in AD patients and rodent models [4]. However, the potential mechanisms of acupuncture on AD remain unclear.

Neuroplasticity is the ability of the brain's response to intrinsic or environmental demands and reorganizing its structure and function, which is engaged in brain development, learning, and self-healing of neural injuries [5]. Neurogenesis, dendritic remodeling and synapse turnover, and modulation of the structure and function of neuronal networks were involved in this physiological process [5–7]. It was reported that neural plasticity dysfunction may contribute to brain network disruption in AD [8]. Abnormal functional connectivity was demonstrated to be a candidate biomarker for AD, and its severity correlates with clinical disease severity in AD [9]. Previous studies have revealed abnormal large-scale functional brain networks in AD patients, which include the default mode network (DMN) [9], the salience network (SN), frontal parietal network (FPN) [10], visual network (VN) [11], sensorimotor network (SMN) [12], and auditory network (AN) [13]. We previously found that enhanced functional connectivity (FC) in some regions of the DMN caused by the acupuncture stimulation of Hegu (LI4) and Taichong (LR3) might be associated with improvement of cognitive function in AD patients [14]. According to traditional Chinese medicine, both Hegu (LI4) and Taichong (LR3) are known as hubs for internal and external energy gathering and transforming. And the combination stimulus of Hegu (LI4) and Taichong (LR3) is defined as “the four gates,” which is usually applied to promote the circulation of “Qi” and blood throughout the whole body [14]. Besides the DMN, the effect of acupuncture on other large-scale resting-state networks remains unknown in AD.

Herein, we aim to explore the effect of combination stimulus of Hegu (LI4) and Taichong (LR3) on other resting-state networks including the salience network (SN), frontal parietal network (FPN), visual network (VN), sensorimotor network (SMN), and auditory network (AN) in the AD patients. Our finding could enrich the understanding of mechanisms of acupuncture on AD.

## 2. Materials and Methods

### 2.1. Subjects

This study was approved by Xuanwu Hospital Medical Ethics Committee of Capital Medical University. Fourteen patients diagnosed with AD were recruited from Xuanwu Hospital of Capital Medical University and underwent professional and complete physical and neurological examinations, standard laboratory tests, and specific neuropsychological evaluations. The diagnosis of AD fulfilled the Diagnostic and Statistical Manual of Mental Disorders (Fourth Edition) criteria [15] for dementia and the National Institute of Neurological and Communicative Disorders and Stroke/Alzheimer's Disease and Related Disorders Association (NINCDS-ADRDA) criteria [16] for clinically probable AD. Subjects with AD have a global score of clinical dementia rating scale (CDR) of 1 or 2 [17]. Fourteen healthy controls (HCs) with normal cognitive function matched by age, gender, and educational level were included as a healthy control (HC) group for neuroimaging comparisons.

Individuals with psychosis, stroke, tumors, trauma, severe hypertension, epilepsy, substance abuse, mental retardation, or contraindications for MRI (cardiac defibrillators, pacemakers, electromagnetic system implants, mechanical heart valves or vascular clips, cochlear implants, or claustrophobia) were excluded. All participants provided their written informed consent before being involved in the study.

### 2.2. Acupuncture Timeline

**Figure 1:**
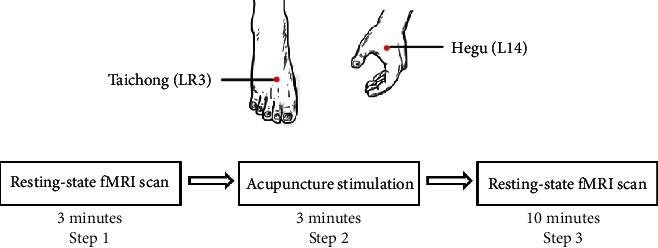
Timeline of fMRI acquisition and acupuncture.

Cloud & Dragon brand (Cloud & Dragon Medical Device Co., Ltd, Jiangsu, China) disposable acupuncture needles (size 0.30 × 25 mm) were used. After baseline MRI scans, all participants received manual acupuncture at bilateral Hegu (LI4, located in the hand dorsum) and Taichong (LR3, located in the dorsalis of the foot) (Figure 1 for all point locations). After skin disinfection, acupuncture needles were inserted 10 to 15 mm into the skin. Following needle insertion, manual stimulations such as small, equal manipulations of twirling, lifting, and thrusting were performed on all needles to induce characteristic sensation “de qi” (a composite of sensations including soreness, numbness, distention, heaviness, and other sensations), which is assigned as an essential component for acupuncture efficacy [18]. The process of acupuncture stimulation lasted for three minutes, and then, all needles were withdrawn.

### 2.3. MRI Acquisition

The baseline resting-state MRI data were acquired before the process of acupuncture stimulation. Participants firstly had rest for three minutes and then received manual acupuncture stimulation for three minutes. After the needles were withdrawn, another 10 minutes of resting-state fMRI scans was acquired (Figure 1).

MRI data acquisition was performed on a 3.0 T MR scanner (Siemens, Erlangen, Germany). Foam padding and headphones were used to control head motion and scanner noise. The data scan parameters of resting-state fMRI were as follows: repetition time (TR) = 2,000 ms, echo time (TE) = 40 ms, flip angle (FA) = 90°, matrix = 64 × 64, field of view (FOV) = 240 × 240 mm^2^, slice thickness = 3 mm, slice gap = 1 mm, voxel size = 3.75 × 3.75 × 3 mm^3^, and bandwidth = 2232 Hz/pixel. Rapid collection gradient echo (MP-RAGE) sequence prepared by magnetization method was used to acquire sagittal T1-weighted MR images (TR/TE = 1, 900/2.2 ms; FA = 9°; matrix = 256 × 256; inversion time = 900 ms; slice thickness = 1 mm, no gap; 176 slices).

All image data were analyzed using the Data Processing Assistant for Resting-State fMRI (DPARSFA) [19]. The first 10 volumes of each subject were removed, to make the signal to reach equilibrium and participants' adaptation to the scanning noise. The remaining volumes were corrected for within-scan acquisition time differences between slices, and the images with head movement greater than 2 mm in any direction or head rotation greater than 2° were excluded. To spatially normalize the fMRI data, the realigned volumes were spatially standardized into the MNI space using the EPI template. The functional images were resampled into a voxel size of 3 × 3 × 3 mm^3^. Then, the functional images were smoothed with a Gaussian kernel of 4 × 4 × 4 mm^3^ full width at half maximum (FWHM) to reduce the spatial noise.

### 2.4. Independent Component Analysis (ICA)

The putative resting-state networks analysis was conducted by the group independent component analysis (ICA) of the GIFT software (http://icatb.sourceforge.net, version 1.3i) [20], and the minimum component length (MDL) criterion was used to determine the number of independent components (ICs) [21]. The fMRI data of all participants were concatenated into one group, the temporal dimension of the aggregate dataset was reduced through principal component analysis (PCA), and the separation of data was performed by the IC estimation of the informax algorithm (with time course and spatial maps) [22, 23]. We further compared the stability of IC estimates of image data to account for spatial correlation at order estimated by minimum description length (MDL) criterion, based on the filtered and unfiltered data by using the software package ICASSO. ICs and time courses of each participant were back-reconstructed by GICA [24].

We identified the brain network by visual inspection, which was previously described [25]. The individual-level components were extracted from back-reconstruction and were converted into *z*-scores, which reflect the degree of correlation between the time series of a given voxel and the mean time series of its components. For each network component, the *z*-score of each voxel was defined as the resting-state intranetwork FC.

### 2.5. Statistics Analysis

Continuous variables of demographics and neuropsychological scores were presented as mean ± standard deviation (SD) and compared by Student's *t*-tests or Mann-Whitney *U* test based on distributional properties. Categorical variables were described as percentages and compared by chi-square tests. The statistical tests were two-sided, and a *P* value < 0.05 was considered statistically significant. Analyses were conducted with SPSS version 25.0 (IBM Corp, Armonk, NY, USA).

The statistics analysis of images was performed using Statistical Parametric Mapping software (SPM12; http://www.fil.ion.ucl.ac.uk/spm/software/spm12). First, one-sample *t*-test was used to achieve the brain network *z*-statistic map for each condition (HC_before, HC_after, AD_before, and AD_after). We use a family-wise error (FWE) correction with a threshold of *P* < 0.05 for multiple comparisons. Then, the statistics mask was made by combining the 4 conditions (i.e., HC_before, HC_after, AD_before, and AD_after), which were applied to compare the between-group differences (HC_before vs AD_before) and the interaction effect of acupuncture by group ([AD_before > AD_after] > [HC_before > HC_after], [AD_after > AD_before] > [HC_after > HC_before]). Second, two-sample *t*-test was used to indicate the differences of the whole brain network (*P* < 0.05, FWE correction) between-group difference in AD and HC groups before acupuncture (NC_before vs. AD_before) controlling for gender and age as covariates. Based on the group differences before acupuncture (HC_before vs. AD_before), regions of interest (ROIs) were defined according to the activated clusters. To determine the effect of acupuncture on modulating brain network connectivity, we extracted the *z* values for the regions with different connections. Then, a paired *t*-test was involved in comparing the effects of acupuncture on the HC and AD groups in each ROI. Finally, a general linear model (GLM) [26] of covariance was used to compare the interaction effect (*P* < 0.05, FWE correction) of acupuncture (before vs. after) by group (AD vs. HC). In addition, we calculated the respective simple main effects (i.e., AD_before vs. AD_after and HC_before vs. HC_after).

## 3. Results

### 3.1. Clinical Data and Neuropsychological Test

In the study, both two HCs and two AD patients were excluded from the study due to the head motions (>2 mm and 2°). Then, the clinical information of 24 participants who underwent the MRI scans is shown in Table 1. There was no significant difference of age and gender between the AD patients and HCs. And lower scores of CDR, Montreal Cognitive Assessment (MoCA), and auditory verbal learning test (AVLT) were investigated in AD patients compared with those of HCs (*P* < 0.01).

### 3.2. The Identification of Large-Scale Brain Networks in AD Patients and HCs

**Figure 2:**
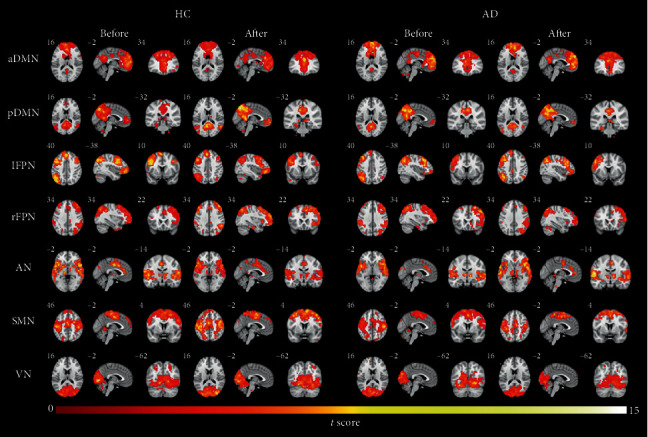
Within-condition anterior DMN (aDMN), posterior DMN (pDMN), left frontal parietal network (lFPN), right frontal parietal network (rFPN), auditory network (AN), sensorimotor network (SMN), and visual network (VN) connectivity patterns identified by independent component analysis (ICA), including patterns for the 2 conditions before acupuncture (i.e., HC_before and AD_before) and 2 conditions after acupuncture (i.e., HC_after and AD_after).

Figure 2 showed some intrinsic network maps for 4 conditions (HC_before, HC_after, AD_before, and AD_after). Besides the DMN, five components of interest including left frontal parietal network (lFPN), right frontal parietal network (rFPN), auditory network (AN), sensorimotor network (SMN), and visual network (VN) were revealed by visual inspection [27].

### 3.3. Comparison of Functional Connectivity of the Large-Scale Resting-State Networks between AD Patients and HCs before Acupuncture

**Figure 3:**
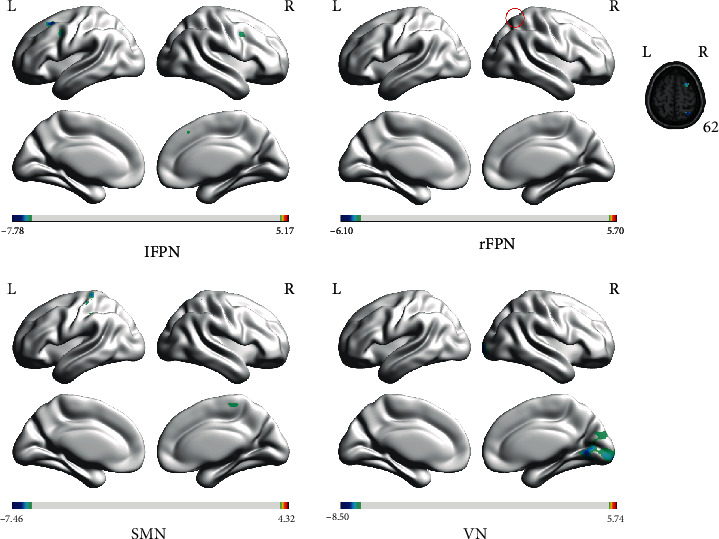
The large-scale resting state networks with different activities of FC between AD patients and HCs before acupuncture (the color scale represents *t* values). FC: functional connectivity; AD: Alzheimer's disease; HCs: healthy controls; lFPN: left frontal parietal network; rFPN: right frontal parietal network; SMN:, sensorimotor network; VN:, visual network; L: left; R: right.

Before acupuncture, except the functional connectivity in the AN without significant difference, the connectivity of other identified networks including lFPN, rFPN, SMN, and VN showed to be decreased in AD patients, compared with those of HCs. Some clusters included the left superior frontal gyrus (SFG), the left middle frontal gyrus (MFG), left precuneus, the left inferior frontal gyrus (IFG), and right MFG located in the lFPN; the right angular gyrus (AG) and right MFG in the rFPN; the left IPL, bilateral postcentral gyrus (PoCG), and right MFG in the SMN; and the right middle occipital gyrus (MOG) of VN and presented to be significantly decreased in AD patients (Figure 3 and Table 2).

### 3.4. The Effect of Acupuncture on These Networks Separately in HCs and AD Patients

**Figure 4:**
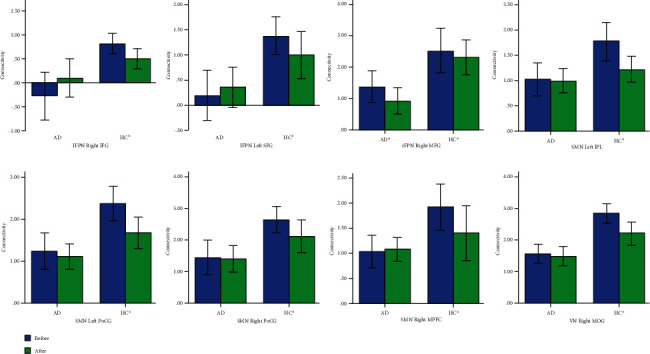
Eight regions of large-scale networks with changes of FC after acupuncture. AD: Alzheimer's disease; HCs: healthy controls; lFPN: left frontal parietal network; rFPN: right frontal parietal network; SMN: sensorimotor network; VN: visual network; IFG: inferior frontal gyrus; SFG: superior frontal gyrus; MFG: middle frontal gyrus; IPL: inferior parietal lobule; PoCG: postcentral gyrus; MOG: middle occipital gyrus.

Then, we explored the associated pattern of the acupuncture's influence on these networks above described, separately in HCs and AD patients. Eight ROIs showed changes related to acupuncture, which included the right IFG and left SFG of lFPN; the right MFG of rFPN; the left IPL, left PoCG, right PoCG, and right MFG of SMN; and the right MOG of VN. Among these ROIs, significantly decreased activity in the right MFG of rFPN (*P* = 0.007) was identified in AD patients, not in the HCs. Significantly decreased FC in the right IFG (*P* = 0.047) and left SFG (*P* = 0.041) of lFPN; the left IPL (*P* = 0.004), left PoCG (*P* = 0.001), right PoCG (*P* = 0.032), and right MFG (*P* = 0.010) of SMN; and the right MOG of VN (*P* = 0.003) were identified in HCs, not in the AD patients. (Figure 4).

### 3.5. The Explorations of the Acupuncture Effect on Large-Scale Resting-State Networks in AD

**Figure 5:**
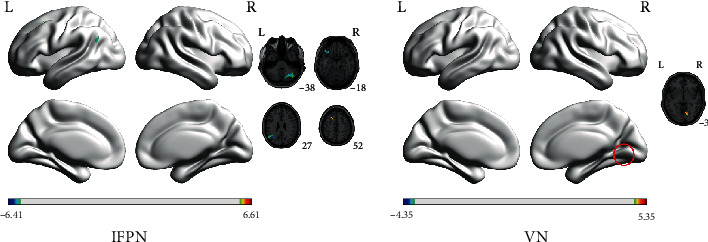
Decreased or increased FC possibly related to acupuncture in some regions of lFPN and VN in AD patients (the color scale represents *t* values). FC: functional connectivity; lFPN: left frontal parietal network; VN: visual network; L: left; R: right.

Figure 5 shows the interaction effect of acupuncture by group, and details of related changes in the FC are shown in Table 3. After controlling for the effect of acupuncture on HCs, decreased FC in the right cerebellum crus I, left IFG, and left AG of lFPN was identified, while the increased FC in the left MFG of IFPN and the right lingual gyrus of VN was found in AD patients.

## 4. Discussion

AD is a progressive and complicated neurodegenerative disease caused by neuronal degeneration and cell loss of the whole brain [28]. And its symptoms included memory loss, disorientation, mood and behavior changes, confusion, unfounded suspicions, and eventually, difficulty speaking, swallowing, and walking. In our study, we found decreased FC in some identified large-scale networks including lFPN, rFPN, SMN, and VN in AD patients, which suggested that AD might be related to changes of FC in larger-scale functional networks responsible for these abnormal behaviors [13, 29–31]. Interestingly, we did not find the significant effect of the combination stimulus of Hegu (LI4) and Taichong (LR3) on all of the identified networks in AD patients. After controlling for the effect of acupuncture on HCs, we found the decreased FC in some regions of the lFPN, while increased FC in some regions of IFPN and VN in AD patients, which might suggest the effect of acupuncture on some restricted regions of specific brain networks selectively in AD. These results revealed the effect of the acupuncture stimulation of Hegu (LI4) and Taichong (LR3) on some large-scale brain networks of AD patients beyond DMN and might suggest the potential mechanism of acupuncture's effect on AD.

The FPN was reported to play an important role in the executive control function and language [32]. Many studies indicated that the functional disconnection and compensation in the FPN might coexist in AD [33]. Zhao et al. found that the superior parietal gyrus (SPG) regions and left paracentral lobule (PCL) of FPN presented increased FC, while the left supramarginal gyrus (SMG) and left inferior parietal (IPL.L) regions showed decreased FC in AD patients [34]. Although our results showed FC reduced within the FPN in AD patients, we found both disrupted and excessive enhanced FC of different regions in lFPN after combination stimulus of Hegu (LI4) and Taichong (LR3). It is possible that during acupuncture, the region was activated to preserve and compensate for losses in function attributable to the degenerative effects of the disease; conversely, the region was reduced to restrain the excessive enhance due to the disease. All these findings might have coincided with the theory of dynamic functional reorganization.

Usually, visual cortices are activated not only by visual stimuli but also by visual mental imaginary and remembering [35]. Previous studies suggested that acupuncture at Taichong (LR3) could specifically perform the bidirectional regulation (excitation and inhibition) on vision-related brain areas [36, 37], which might be a mechanism for treating vision-related diseases. This observation confirmed reduced FC of VN in the healthy olds and further verified that acupuncture stimulus of Taichong (LR3) might specifically regulate the VN. Moreover, complex visual disturbances are observed in AD patients, including constructional and visuoperceptual disorientation, specifically difficulties in searching for objects (figure–ground discrimination), finding their way in familiar surroundings (environmental agnosia) [38, 39]. Many studies found that the reduced FC of ventral/dorsal VN could play a crucial role in the visuospatial disorder of patients with AD pathology [9, 40–42], which may associate with amyloid-beta and neurofibrillary tangle (NFT) aggregation as well as neurodegeneration and axonal damage in visual cortical regions in AD pathology [43]. We found that combination stimulus of Hegu (LI4) and Taichong (LR3) activated FC of some regions in the VN of AD patients, which suggested acupuncture at Hegu (LI4) and Taichong (LR3) might treat visual disturbances in AD.

Many evidences suggested that stimulation at Hegu (LI4) and Taichong (LR3) induced distinct response patterns. For example, it was reported that acupuncture at Hegu (LI4) mainly specifically deactivated right frontal areas [37]. And many researches showed that acupuncture at Taichong (LR3) mainly specifically activated the brain functional network that participates in visual function, associative function, and emotion cognition [37, 44]. This observation identified decreased FC of the rPFN in AD patients and significantly reduced FC of the SMN, lFPN, and VN in the HCs after acupuncture at Hegu (LI4) and Taichong (LR3). These results coincided with previous findings, which suggested that acupuncture at Hegu (LI4) and Taichong (LR3) may regulate the neuroplasticity of specific brain networks to induce therapeutic effects. But we only find acupuncture at Hegu (LI4) and Taichong (LR3) effect in the VN and lFPN in AD patients, after controlling for the effect of acupuncture on HCs. This might be partly explained by pathology and characteristics of AD; that is, the destruction of the SMN and rFPN due to AD might be so severe that acupuncture failed to preserve their integrality. Thus, FC in more regions were significantly changed in HC than AD. Moreover, a relatively small sample size might provide another explanation.

Previous studies have found that acupuncture could modulate the synaptic plasticity in AD patients or animal model [45], by enhancing long-term potentiation (LTP) and long-term depression (LTD) of the hippocampus [46], increasing the synapse number and postsynaptic density thickness [47], and modulating regional spontaneous activity [48, 49]. It is well known that neuroplasticity might contribute to preserve the integrality of brain networks. In this study, the neurophysiology mechanism of this influence on the lFPN and VN of AD patients remains unclear, which remains to be established.

There are still some limitations in our study. First, we did not demonstrate whether the enhancement or reduction was associated with acupoint specificity due to the lack of the sham acupuncture needle. Second, we did not find the correlation between changes of functional connectivity of these networks after acupuncture and behaviors in AD patients. Third, a relatively small sample size was included in this study. Furthermore, despite controlling the sequence of scan and using the ICA method, the influence of the scan lengths on the reliability and similarity of FC would be inescapable [50, 51]. The changes in FC after a few minutes of stimulation may be merely a transient change in reactivity, which might not completely equal to the effect of acupuncture on neuroplasticity. Given that the sustained effect of acupuncture on the network is unclear, in the future, we will design a longitudinal study to trace these patients at different time points and explore the alteration of brain network in patients with AD after acupuncture for a period time. Therefore, future longitudinal studies with a much larger sample are warranted to elucidate the progressive functional changes caused by acupuncture in AD patients and its relationship with the clinical performances.

## 5. Conclusion

In conclusion, by using the ICA method, our study found that the activity of large-scale brain networks in AD patients could be mediated by the acupuncture stimulation on Hegu (LI4) and Taichong (LR3). These findings have important implications for the underlying neurobiology of AD and the effect of acupuncture on the disease, which might have some reference values for the interpretation of the therapeutic effects of acupuncture at Hegu (LI4) and Taichong (LR3) on AD in the future.

## Figures and Tables

**Table 1:** Characteristics of the patients with AD and HCs.

	AD (*N* = 12)	HCs (*N* = 12)	*P* value
Gender, female/male	7/5	7/5	1.00
Age (year)^∗^	67.58 ± 9.05	64.83 ± 6.94	0.259
Education (year)^∗^	9.17 ± 3.19	11.58 ± 4.60	0.178
Course (month)^∗^	11.5 ± 5.16	—	—
MoCA^∗^	14.83 ± 3.41	27.75 ± 0.62	<0.01
CDR^∗^	1.04 ± 0.33	0.00 ± 0.00	<0.01
AVLT (immediate)^∗^	12.17 ± 3.61	25.50 ± 5.39	<0.01
AVLT (short time)^∗^	2.58 ± 1.56	10.83 ± 3.01	<0.01
AVLT (long time)^∗^	3.25 ± 1.82	12.92 ± 1.73	<0.01

^∗^Mean ± SD. AD: Alzheimer's disease; HCs: healthy controls; MoCA: Montreal Cognitive Assessment; CDR: clinical dementia rating scale; AVLT: auditory verbal learning test; immediate: immediate recall of learning verbal; delayed: delayed recall of learning verbal; recognition: recognition of learning verbal; SD: standard deviation.

**Table 2:** Comparison of large-scale networks before acupuncture between AD patients and HCs.

Networks	Brain regions	MNI coordinate			BA	Cluster size	*t* score
		*x*	*y*	*z*			
lFPN	Left SFG	-30	60	21	10	44	5.77
	Left MFG	-48	9	42	8	88	6.53
	Left precuneus	-36	-78	36	19	34	4.65
	Right IFG	45	6	39	9	37	5.73
	Right MFG	3	24	48	8	28	5.28
	Left SFG	-24	15	54	8	31	7.79

rFPN	Right AG	42	-60	54	40	63	6.1
	Right MFG	30	9	60	6	38	5.19

SMN	Left IPL	-42	-30	42	40	36	4.27
	Left PoCG	-36	-30	69	3	121	7.46
	Right PoCG	48	-36	60	40	63	5.35
	Right MFG	3	-27	63	6	57	4.94

VN	Right MOG	21	-99	0	18	752	8.5

FC: functional connectivity; AD: Alzheimer's disease; HCs: healthy controls; BA: Brodmann area; MNI: Montreal Neurological Institute; *x*, *y*, and *z*: coordinates of primary peak locations in the MNI space; lFPN: left frontal parietal network; rFPN: right frontal parietal network; SMN: sensorimotor network; VN: visual network; SFG: superior frontal gyrus; MFG: middle frontal gyrus; IFG: inferior frontal gyrus; AG: angular gyrus; IPL: inferior parietal lobule; PoCG: postcentral gyrus; MOG: middle occipital gyrus.

**Table 3:** Brain regions with decreased or increased FC possibly related to acupuncture in the AD patients.

Networks	Brain regions	MNI coordinate			BA	Cluster size	*t* score
		*x*	*y*	*z*			
	Decreased: (AD_before > AD_after) > (HC_before > HC_after)						
lFPN	Right cerebellum crus I	36	-63	39	—	111	5.37
	Left IFG	-45	30	-15	47	48	5.81
	Left AG	-39	-63	33	39	90	6.42

	Increased: (AD_after > AD_before) > (HC_after > HC_before)						
lFPN	Left MFG	-24	18	51	8	30	5.68
VN	Right LG	15	-72	-6	18	29	4.73

FC: functional connectivity; AD: Alzheimer's disease; HCs: healthy controls; BA: Brodmann area; MNI: Montreal Neurological Institute; *x*, *y*, and *z*: coordinates of primary peak locations in the MNI space; lFPN: left frontal parietal network; VN: visual network; IFG: inferior frontal gyrus; AG: angular gyrus; MFG: middle frontal gyrus; LG: lingual gyrus.

## Data Availability

The clinical and image data used to support the findings of this study are available from the corresponding author upon request.
